# Fra-1 and c-Fos N-Terminal Deletion Mutants Impair Breast Tumor Cell Proliferation by Blocking Lipid Synthesis Activation

**DOI:** 10.3389/fonc.2019.00544

**Published:** 2019-06-19

**Authors:** Ana Cristina Racca, César Germán Prucca, Beatriz Leonor Caputto

**Affiliations:** Departamento de Química Biológica Ranwel Caputto, Facultad de Ciencias Químicas, Centro de Investigaciones en Química Biológica de Córdoba (CIQUIBIC-CONICET), Universidad Nacional de Córdoba, Córdoba, Argentina

**Keywords:** c-Fos, Fra-1, phospholipids, CDP-diacylglycerol synthase, phosphatidylinositol synthase

## Abstract

Tumor cells require high rates of lipid synthesis to support membrane biogenesis for their exacerbated growth. The only two proteins known that activate phospholipid synthesis are Fra-1 and c-Fos, two members of the AP-1 family of transcription factors. These proteins that are overexpressed in human breast malignant tumors increase the rate of phospholipid synthesis at the endoplasmic reticulum through a mechanism independent of their nuclear function. The aim of this study was to inhibit breast tumor cell proliferation by modulating c-Fos and Fra-1 and regulate membrane biogenesis by controlling lipid synthesis rates. The molecular mechanism by which Fra-1 and c-Fos activate phospholipid synthesis was examined. Both proteins physically associate with the rate limiting enzyme CDP-DAG synthase through their N-terminus domain and activate it through their basic domain; neither protein associates to or activates the enzyme phosphatidylinositol synthase as determined through *in vitro* enzymatic reactions and FRET experiments. The N-terminus domain of both proteins act as negative dominant peptides that physically associate with CDP-DAG synthase but do not activate it. Proliferation of MDA-MB231 and 4T1 cells was impaired *in vitro* after inducing them to proliferate in the presence of the negative dominant peptides derived from Fra-1 and c-Fos. When tumors generated in Balb/c mice with the breast tumor cell line 4T1 were treated with these negative dominant peptides, a significant reduction in tumor growth was observed. Consequently, these Fra-1 and c-Fos negative dominant peptides can be exploited as a new therapeutic strategy to impair breast tumor cell proliferation.

## Introduction

Breast cancer is the most frequent cancer among women (25% of all cancers) and the main cause of cancer deaths in less developed countries (1). According to the World Health Organization, its incidence is increasing worldwide. Although prevention and early detection significantly reduce death risk, they do not eliminate most breast cancers that develop in low- and middle-income countries where these are diagnosed at late stages and generally result in patient death. Breast cancer is the fifth cause of cancer deaths after lung (1.6 million deaths), liver (745,000 deaths), stomach (723,000 deaths), and colorectal (694,000 deaths) cancers (2). So, development of new therapeutic strategies targeted to inhibit breast tumor cell proliferation in established tumors is essential.

Fra-1 and c-Fos are two well-known oncogenes, members of the AP-1 family of transcription factors (3, 4). Their nuclear activity and overexpression in diverse tumors has been widely reported (5). Fra-1 is overexpressed in breast, lung, colon, esophagus, bladder, thyroid and hepatocellular tumors, among others (5–13). Moreover, Fra-1 has been implicated in cervical cancer stem cell radio-resistance (14), in the malignant progression of esophageal squamous cell carcinoma through MAPK/MEK/ERK/FRA-1 pathway activation (15), in epithelial-mesenchymal transition regulation of colorectal cancer cells (16, 17), in cancer stem cell formation from non-stem cells in a PKCα-dependent activation of Fra-1 (18), and in the inhibition of p53-dependent apoptosis in lung cancer (19). However, despite having been studied for almost 30 years, the cellular consequences of c-Fos and Fra-1 expression upon mitogen stimulation have not been completely elucidated.

In breast tumors, the transcription factors Fra-1 and c-Fos are also expressed in the cytoplasm where they activate phospholipid synthesis (20). Phospholipids are the quantitatively most important components of cell membranes thus making their production essential to sustain cell proliferation, differentiation and growth. Tumor cells are characterized by their exacerbated growth and so, demand high rates of phospholipid synthesis to support increased membrane biogenesis. Fra-1 and c-Fos show a similar capacity to activate phospholipid synthesis in proliferating breast tumor cells in culture (MDA-MB231 and MCF-7 cells) and in human breast tumor tissues. In more than 200 malignant human breast tumors examined (Invasive ductal carcinoma, Medullary carcinoma, Phyllodes sarcoma, Mucinous carcinoma, lobular carcinoma *in situ* and squamous cell carcinoma) 95% of both proteins were significantly overexpressed and 100% had either Fra-1 or c-Fos overexpressed contrasting with their undetectable levels in normal tissue. Fra-1 was found mainly in the cytoplasm: 69% of tumor samples showed only cytoplasmic Fra-1, while the remaining 31% contained both nuclear and cytoplasmic Fra-1. c-Fos was also preferentially in the cytoplasm of the tumor samples: 100% showed cytoplasmic c-Fos and 63% also contained nuclear c-Fos. In all cases, Fra-1 and c-Fos localized with the Endoplasmic Reticulum (ER) marker calnexin where they both participate in the bulk phospholipid synthesis. Silencing Fra-1 and c-Fos simultaneously and, more importantly, blocking the cytoplasmic activity of these proteins with specific antibodies significantly inhibits lipid synthesis activation and cell proliferation in MDA-MB231 cells (20). Based on these results, cytoplasmic c-Fos and Fra-1 deserve to be considered as potential targets to control proliferation of breast cancer cells. To seek a therapeutic application of these results, we determined the molecular mechanism by which these proteins activate phospholipid synthesis. For c-Fos to promote activation, it must associate with enzymes of the lipid synthesis pathway in the ER, through its N-terminal domain (aa 1–138) and increases their catalytic activity through its basic domain (aa 139–159) (21). c-Fos association to the ER is regulated by the phosphorylation status of its tyrosine-residues #10 and #30 (22).

c-Fos activates several and the same enzymes in different cell types. Specifically, c-Fos activates 1-acylglycerol-3-phosphate acyltransferase, CDP-diacylglycerol synthase (CDS) the rate-limiting enzyme of the phosphoinositide synthesis pathway, phosphatidylinositol 4-kinase II α (PI4KIIα) and Lipin1β which drives phosphatidic acid into the Kennedy pathway. However, c-Fos does not modify the activity of phosphatidylserine synthases 1 and 2, phosphatidylinositol synthase (PIS) or PI4KIIβ (21, 23, 24). A similar effect is observed for glycosphingolipid synthesis, in which c-Fos activates glucosylceramide synthase but does not affect glucosylceramide galactosyltransferase 1 or lactosylceramide sialyltransferase 1 (25). Fra-1 also activates the overall synthesis of phospholipids and has been shown to activate Lipin 1β (20, 26). However, the mechanism by which Fra-1 activates phospholipid synthesis still remains unexplored.

Herein, we examine the mechanism by which Fra-1 activates phospholipid synthesis in a breast tumor cell model. Two enzymes were examined; one that was previously shown to be activated by c-Fos (CDS) and one whose activity is not modified by c-Fos (PIS). We observed that Fra-1 associates to CDS1 and activates total CDS, whereas it neither associates to nor activates PIS.

Results shown herein indicate that Fra-1 and c-Fos can be the foundation for a novel therapeutic strategy to inhibit breast tumor growth by impairing membrane biogenesis.

## Materials and Methods

### Cell Culture

MDA-MB231 and 4T1 cells from ATCC were cultured as indicated by the supplier. *Mycoplasma* control was regularly performed. Cultured cell quiescence was achieved after culturing 48 h using DMEM without FBS and phenol red. Cells were induced to re-enter growth by adding 20% FBS.

### Proliferation Assay

Transfections were performed following the manufacturer's instructions in 24 multi-well plates using Lipofectamine 2000 (Invitrogen) plus 400 ng of pTQ2-Fra-1-NA or pTQ2-c-Fos-NA, or a mixture of 200 ng of each. Control cells received 400 ng of the pTQ2 vector. Twenty-four hours after transfection, cells were cultured in serum-free media for 48 h to achieve quiescence (0 h), then 20% FBS was added to the culture medium (mitotic stimulus) and cells cultured for an additional 30 h. Turquoise fluorescence was used to determine the number of transfected cells; total cell number by propidium iodide staining and double blind cell counting in each condition was performed in at least 20 fields per experiment. By subtracting the number of transfected cells from total cells, the number of non-transfected cells was determined at 0 h and at 30 h. Cell proliferation was calculated as the ratio between the number of cells at 30 h/0 h.

### Expression and Purification of Recombinant Proteins

Recombinant proteins were expressed as His-tagged recombinant proteins in the BL21 strain of *Escherichia coli* and purified as described previously (27, 28) by nickel affinity chromatography. Cells were re-suspended in lysis buffer containing 6 M urea for wild type proteins or without 6 M urea for deletion mutants and lysed using the Avestin Emulsiflex C3. Cell homogenates were run through a HisTrap HF column (GE Healthcare Life Sciences), washed thoroughly and proteins purified using an Äkta Purifier 10 (GE Healthcare Life Sciences). Elution buffer (0.5 M NaCl, 20 mM Tris-HCl pH 8, 500 mM imidazole) with Urea (6M) was used for c-Fos and Fra-1, and without urea for Fra-1 deletion mutants and c-Fos-NA. Final imidazole concentration in the eluted proteins was 171 mM.

### Enzyme Activity Determinations

Reactions performed in a final volume of 80 μL contained, 100 μg of quiescent MDA-MB231 cell homogenate protein in 50 mM Tris-HCl, pH 8 (enzyme source). For enzyme activity assays in animal excised tumors, homogenates were prepared in water containing protease inhibitors using a Potter-Elvehjem Tissue Grinder, prior to sonication. Total CDS activity was assayed as described by Lykidis et al. (29) with or without the recombinant c-Fos, Fra-1 or its deletion mutants Fra-1-NA, Fra-1-NB, Fra-1-BC, as stated in each case, suspended in 3 μL of elution buffer or an equal volume of elution buffer for control reactions.

Total PIS activity reactions were as described by Lykidis et al. (29) in the presence of c-Fos or Fra-1 suspended in 3 μL of elution buffer or with elution buffer for control reactions.

### Colocalization Analysis

MDA-MB231 cells were co-transfected with pEGFP-Fra-1 or pEGFP-c-Fos and both NA deletion mutants fused to the HA tag or the control with the empty vector. Immunofluorescence was performed using anti-HA (mouse Sigma #H9658 1:100) and anti-calreticulin (rabbit Thermo Scientific #PA3-900 1:800), secondary antibodies: anti-mouse 546 and anti-rabbit 643 (1:600). A spectral Olympus (Center Valley, PA) FV 1200 confocal laser scanning microscope with a plan apochromatic 60x (SC), numerical aperture 1.4, oil immersion objective lens zoomed digitally 5x was used. Mander's colocalization coefficient M1 (proportion of Fra-1 or c-Fos present in the ER) with threshold was used to quantify colocalization using the Fiji software and the Jacop plugin.

### FRET Analyses

FRET experiments were performed as described previously (21). Cells transfected with GFP derivatives-coupled enzymes and proteins/peptides as indicated were arrested for 48 h. CDS1 and PIS (pSYFP2-N1) and pTQ2-c-Fos were previously used (21). Wild type Fra-1 and truncated mutants of Fra-1 were in pTurquoise2-N1. Synthesis of endogenous c-Fos and Fra-1 was impaired using 50 μg/mL of cycloheximide 1 h prior to inducing cells to re-enter growth with FBS. Fifteen minutes later, cells were fixed with 4% paraformaldehyde for 10 min at room temperature. Mounted Coverslips were visualized using an Olympus FV300 confocal laser scanning microscope with a plan apochromatic 63x, numerical aperture 1.4, oil immersion objective lens zoomed digitally 2x. FRET determinations were by measuring sensitized emission (30, 31). Images analyzed with Fiji (NIH, USA) were pseudo-colored to illustrate the calculated efficiencies distribution in cells.

### Treatment of Breast Tumors *in vivo*

This study was carried out in accordance with the recommendations of the ethics committee of experimental protocols for the use of animals (Facultad de Ciencias Químicas, Universidad Nacional de Córdoba). The protocol was approved by the ethics committee of experimental protocols for the use of animals (Facultad de Ciencias Químicas, Universidad Nacional de Córdoba. Resolution N° 2102). The mouse-4T1 breast tumor model was used as described in Pulaski et al. (32). Ten days after inoculation, animals received an intra-tumor injection at 3-days intervals of either Fra-1-NA + c-Fos-NA-containing liposomes (treated) or empty liposomes (controls). Liposomes were prepared by the lipid film hydration method (33, 34). Briefly, diphosphatidylcholine, diphosphatidylserine, and cholesterol (Avanti Polar Lipids 850355P, 840037P, 700000P) (70:10:20 ratio) were dissolved in chloroform (5 mg/mL) and evaporated under nitrogen. Hydration was performed by addition of the peptide solution or vehicle (peptide elution buffer) followed by five cycles of incubation at 55 °C and vortexing. Liposome size was determined by light scattering. A total amount of 22.5 nmol of peptides/mouse in 5 injections delivered 3 times a week was used in low dose experiments. Each injection contained 2.5 nmol of Fra-1-NA (30 μg) plus 2 nmol of c-Fos-NA (30 μg) contained in liposomes re-suspended in 50 μL of peptide elution buffer for the first 2 injections and in 100 μL for the last 3 injections. Empty liposomes (controls) were re-suspended in elution buffer as for the treated animals. For high dose experiments, total peptide administered was 45 nmol/mouse in 5 injections delivered 3 times a week, each containing 5 nmol of Fra-1-NA (60 μg) plus 4 nmol of c-Fos-NA (60 μg) re-suspended in 50 μL of elution buffer for the first 2 injections and in 100 μL for the last 3 injections. Injection volume was varied due to the variation of the tumor volume.

When each mouse harbored 2 tumors, animals received a 2 × 10^5^ 4T1 cell inoculation into each fat pad and upon tumor generation, one tumor was subjected to the high dose treatment protocol described above whereas the other tumor of the same animal was treated as described for the control group. In all cases, tumors (length, width) were measured using a caliper. For western blot experiments, tumors were excised and frozen (−80°C).

### Western Blotting

Using sonication, tumor tissues were processed in ice-cold RIPA buffer (150 mM NaCl, 1% Triton, 50 mM Tris-HCl pH 6.8, 0.5 mM EDTA, SDS 0.01%) plus protease inhibitor (Roche) and centrifuged for 10 min at 12,000 g. Protein extracts were separated on a 12% SDS-PAGE and transferred to nitrocellulose membrane (Pall Corporation). After blocking with 5% non-fat milk in 1X PBS—0.05% Tween-20, membranes were incubated with primary antibodies (mouse monoclonal anti-α-tubulin, Abcam ab7291; 1:5,000 and anti-PCNA, Santa Cruz PC10; 1:250) overnight at 4°C. After washing, blots were incubated with donkey anti-mouse IRDye IgG secondary antibody (LI-COR Bioscience, Lincoln, NE, USA; 1:25,000) at room temperature for 1 h. Immuno-detection was performed using ODYSSEY Infrared Imaging System (LI-COR Bioscience).

## Results

### Fra-1 and c-Fos Activate CDS but Not PIS

Previous results from human breast tumors show that 100% of these overexpress either Fra-1 or c-Fos or both oncoproteins; furthermore, non-fixed samples showed activated phospholipid synthesis with respect to control human breast tissue (20). To evaluate the potential therapeutic use of peptides derived from these proteins, we determined if these two proteins share a common mechanism to activate the synthesis of phospholipids, by evaluating their capacity to regulate two enzymes of the pathway of phospholipid synthesis.

**Figure 1:**
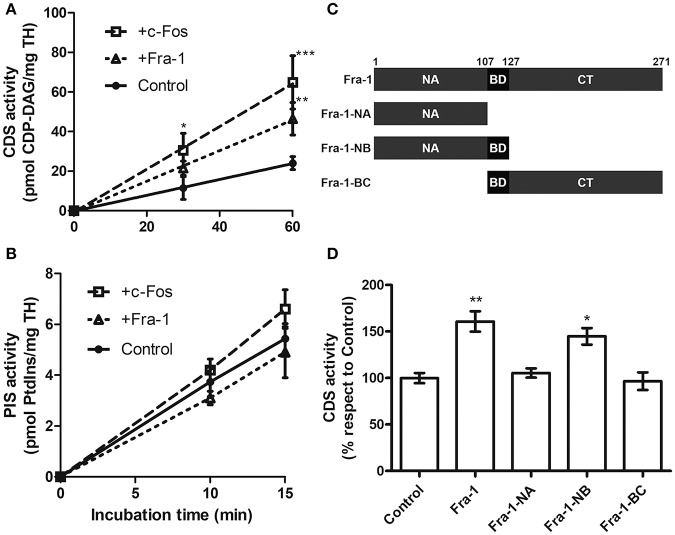
Fra-1 activates CDS through its NB domain. **(A,B)** Total homogenate (TH) from quiescent MDA-MB231 cells was used as enzyme source to evaluate total CDS and PIS activity in the presence of Fra-1 (0.5 ng of recombinant Fra-1 per ug of TH; short dashed line) or c-Fos (0.5 ng of recombinant c-Fos per ug of TH; long dashed line) in comparison with the controls that contained only the vehicle used to re-suspend Fra-1 or c-Fos (continuous line). Both c-Fos and Fra-1 significantly activate CDS **(A)** at the incubation times examined whereas neither of them significantly modified PIS activity **(B)**. **(C)** Schematic representation of the deletion mutants of Fra-1. The numbers on the top of the scheme indicate the amino acid position. Fra-1-NA: N-terminus (aa 1–107), Fra-1-NB: N-terminus plus basic domain (aa 1–127), Fra-1-BC: basic domain plus the C-terminus (aa 108–271). **(D)** CDS activity was evaluated in the presence of recombinant Fra-1 or of its deletion mutants. Only Fra-1-NB activates the enzyme to similar levels as those obtained with the full-length version, whereas Fra-1-NA that lacks the BD or Fra-1-BC that lacks the NA do not retain this activating capacity. ^*^p < 0.05; ^**^p < 0.01; ^***^p < 0.001 (Two-way ANOVA in **A,B** and 1 way ANOVA in **D**). Results are the mean CDS activity ± SEM of three independent experiments performed in triplicate.

To assess the capacity of Fra-1 to activate the enzymes CDS and PIS *in vitro*, total homogenate (TH) from quiescent MDA-MB231 cells was used as enzyme source. Total CDS activity is linear up to 60 min of incubation (Figure 1A) whereas PIS activity is linear up to 15 min of incubation (Figure 1B). At linearity, CDS activity increased in the presence of 0.5 ng of recombinant Fra-1/μg of TH in comparison with the control. By contrast, PIS activity was not significantly modified by the addition of Fra-1 to the assay as compared to the control (Figure 1B). For PIS, increasing concentrations of recombinant Fra-1 were also tested with no changes under any of the experimental conditions evaluated [0–3 ng of Fra-1/μg TH (Supplementary Figure 1)]. Essentially the same results were found when recombinant c-Fos (0.5 ng/μg of TH) was assayed (Figures 1A,B).

### The NB Deletion Mutant of Fra-1 Activates CDS

To determine which domains of Fra-1 are involved in CDS activation, CDS was assayed in the presence of the deletion mutants of Fra-1 schematized in Figure 1C: Fra-1-NA (aa 1–107), Fra-1-NB (aa 1–127) and Fra-1-BC (aa 108–271). Fra-1-NB activates CDS to comparable levels as full-length Fra-1 whereas no activation was observed with Fra-1-NA or Fra-1-BC (Figure 1D). Note that Fra-1-NB is the only mutant assayed that contains both the NA and the BD domains of Fra-1.

### Fra-1 Physically Associates With CDS1 but Not With PIS

**Figure 2:**
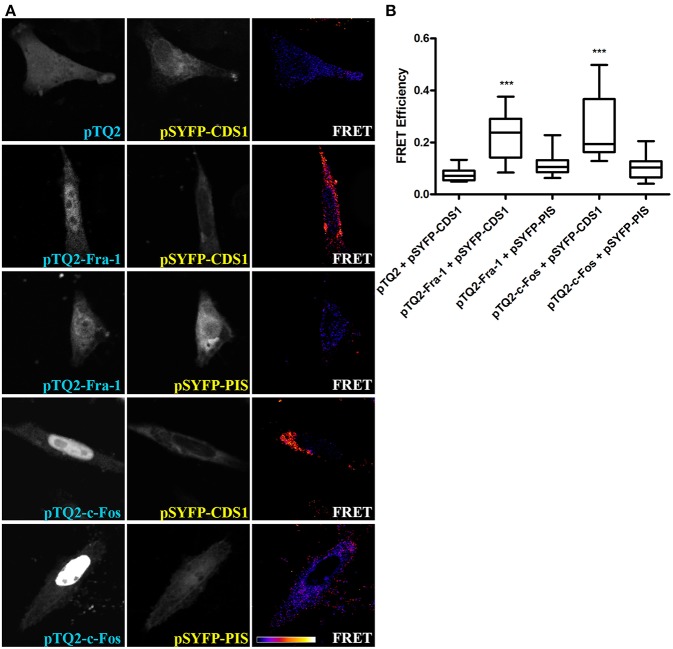
Fra-1 and c-Fos physically associate with CDS1 but not with PIS. **(A)** MDA-MB231 cells were co-transfected to express pTQ2 and pSYFP-CDS1 (first row) as a negative control, pTQ2-Fra-1 and pSYFP-CDS1 (second row), pTQ2-Fra-1 and pSYFP-PIS (third row), pTQ2-c-Fos and pSYFP-CDS1 (fourth row) or pTQ2-c-Fos and pSYFP-PIS (last row), and examined by confocal microscopy; for FRET efficiency quantification, the sensitized emission method was applied. The first column shows tagged Fra-1 or c-Fos expression, the second, the expression of the tagged enzyme and the third column shows FRET efficiency; the scale bar on the bottom row shows an increasing FRET efficiency scale from violet (no FRET) to white. **(B)** FRET efficiency quantification is depicted as boxplot graphs representing the medians (central horizontal bars), the 25–75th percentile interquartile range (box limits), and the lowest and highest values (whiskers). ^***^p < 0.001 (Kruskal-Wallis test, Dunn's post-test). One representative experiment of three performed that gave essentially the same results, is shown.

FRET experiments were performed to evaluate if Fra-1 physically interacts with CDS1 or PIS. MDA-MB231 cells were co-transfected with constructs encoding full length Fra-1 fused to a donor FRET-pair (pTQ2) and each enzyme assayed, CDS1 or PIS, fused to an acceptor FRET-pair (pSYFP). Results show that Fra-1 associates with CDS1 (Figure 2A, row 2) but not with PIS (Figure 2A, row 3). Cells co-transfected with the pTQ2 vector and CDS1 fused to pSYFP show negative FRET values (negative control) (Figure 2A, row 1). Positive FRET values were also found with tagged c-Fos and CDS1 but not with tagged c-Fos and PIS (Figure 2A, rows 4 and 5). Quantification shows statistically significant FRET efficiencies with CDS1 when compared to negative controls whereas neither Fra-1 nor c-Fos shows positive FRET efficiencies when assayed with PIS (Figure 2B). These results evidence that Fra-1 and c-Fos physically associate with the enzyme (s) they activate, like CDS1, but not with those they do not activate, such as PIS.

### Fra-1 Associates With CDS1 Through Its N-terminus Domain

**Figure 3:**
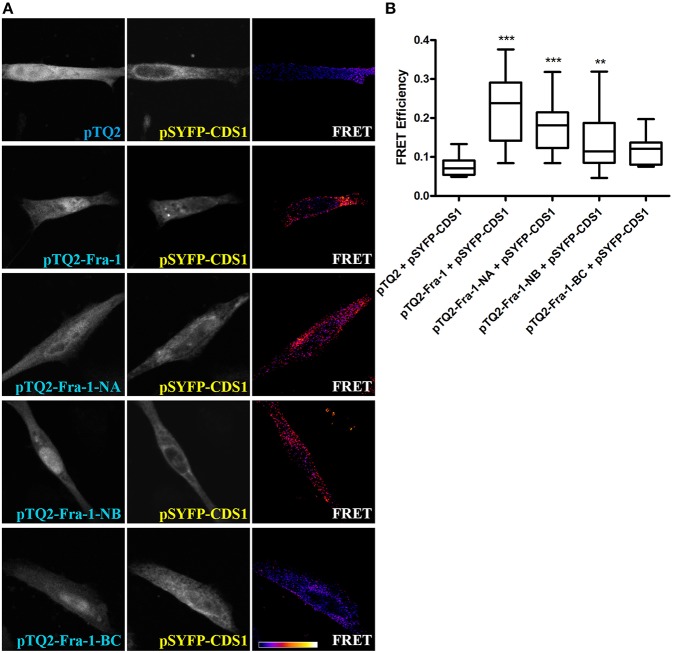
The N-terminus domain of Fra-1 physically associates with CDS1. **(A)** MDA-MB231 cells were co-transfected to express the negative control pTQ2 and pSYFP-CDS1 (first row), pTQ2-Fra-1 and pSYFP-CDS1 (second row), pTQ2-Fra-1-NA and pSYFP-CDS1 (third row), pTQ2-Fra-1-NB and pSYFP-CDS1 (fourth row) or pTQ2-Fra-1-BC and pSYFP-CDS1 (last row), and were examined by confocal microscopy. FRET quantification was performed by the sensitized emission method. The first column shows the expression of tagged Fra-1 or of its deletion mutants, the second of the tagged enzyme and the third column shows FRET efficiency. The scale bar on the bottom row of the right column shows an increasing FRET efficiency scale from violet (negative) to white (maximum). **(B)** FRET efficiency quantification depicted as boxplot graphs representing the medians (central horizontal bars), the 25–75th percentile interquartile range (box limits), and the lowest and highest values (whiskers). ^**^p < 0.01; ^***^p < 0.001 (Kruskal-Wallis test, Dunn's post-test). One representative experiment of three performed, which gave essentially the same results, is shown.

To determine which domain (s) of Fra-1 is/are involved in physical association with CDS1, we performed FRET experiments with the three Fra-1 deletion mutants Fra-1-NA, Fra-1-NB and Fra-1-BC (Figure 1C). MDA-MB231 cells were co-transfected with constructs codifying for Fra-1 or its deletion mutants fused to pTQ2 and CDS1 fused to pSYFP. The mutants Fra-1-NA and Fra-1-NB show positive FRET efficiencies with CDS1, similar to that observed with full-length Fra-1 and statistically different from the negative control (pTQ2 and CDS1 fused to SYFP) (*p* < 0.001 and *p* < 0.01, respectively) (Figures 3A,B). By contrast, Fra-1-BC that lacks the N-terminus domain, does not associate with CDS1 showing similar FRET efficiency to that of the negative control (*p* > 0.05) (Figure 3A, row 5 and Figure 3B). These experiments evidence that Fra-1 associates to CDS1 through its N-terminus domain and that the BD domain is not essential for this association.

### Colocalization of Fra-1 and c-Fos With the ER Marker Calreticulin Decreases in the Presence of Fra-1-NA and c-Fos-NA

Previous reports indicate that Fra-1 and c-Fos colocalize with the ER marker calnexin in human breast tumor samples, and in MDA-MB231 and MCF7 breast tumor cell lines (20). Herein, we quantified the colocalization between Fra-1 or c-Fos and the ER marker calreticulin in the absence or in the presence of Fra-1-NA plus c-Fos-NA deletion mutants by determining Mander's colocalization coefficient M1. For this, MDA-MB231 cells were co-transfected with pEGFP-Fra-1 or pEGFP-c-Fos and the N-terminal deletion mutants fused to the HA tag, that is Fra-1-NA-HA and c-Fos-NA-HA or the control with the HA plasmid. GFP signal was directly detected whereas HA and calreticulin were detected by indirect immunofluorescence using specific antibodies. The results in Supplementary Figure 2 show that Mander's colocalization coefficient M1 that is the proportion of Fra-1 or c-Fos localized in the ER compartment, decreases in the presence of Fra-1-NA and c-Fos-NA.

### The N-terminus Domain of Both Fra-1 and c-Fos Inhibit MDA-MB231 and 4T1 Cell Proliferation

**Figure 4:**
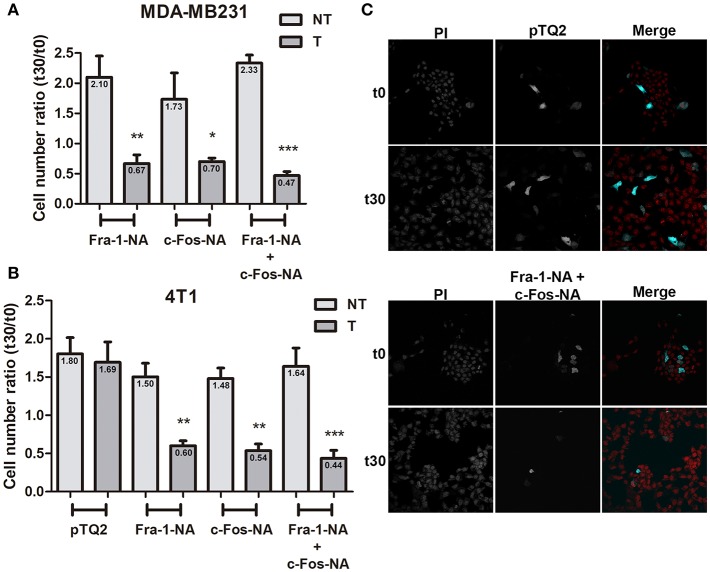
Fra-1-NA and c-Fos-NA inhibit MDA-MB231 and 4T1 cell proliferation. MDA-MB231 **(A)** or 4T1 cells **(B)** were transfected to express pTQ2-Fra-1-NA, or pTQ2-c-Fos-NA, or pTQ2-c-Fos-NA + pTQ2-Fra-1-NA. The number of cells expressing the turquoise fluorescent protein (T: dark gray bars) and the number of non-transfected (NT: light gray bars) cells (propidium iodide stained cells—turquoise cells) present in the same well were counted at 0 h (arrested cells: t0) and 30 h after a mitotic stimulus (20% fetal bovine serum: t30). The ratio between the number of T or NT cells at t30 and t0 is shown and the mean indicated in each bar. Results are expressed as mean ± SEM (*n* = 3). ^*^p < 0.05; ^**^p < 0.01 ^***^p < 0.001 (ANOVA, Bonferroni post-test). **(C)** Representative images of 4T1 cells at t0 (arrested cells) and t30 (after 30 h of a mitotic stimulus) of the control condition transfected with the pTQ2 vector (upper panel) and the condition transfected with pTQ2-c-Fos-NA + pTQ2-Fra-1-NA (lower panel). Total cells stained with PI (left column), and transfected cells (middle column) are shown in black and white and the colored merge in right column. PI: propidium iodide (red), TQ2 (cyan). Original Magnification 400×.

Since Fra-1 and c-Fos physically associate with CDS1 through their N-terminus domain and activate it through their BD domain, and both are overexpressed in breast tumor tissues in comparison with their undetectable levels in normal tissue (20), we hypothesized that the NA domains of both proteins, could function as negative dominants of the cytoplasmic activity of c-Fos and Fra-1 by associating with the enzymes that the full-length proteins activate, but without activating them. So, it is expected that the presence of these deletion mutants, will promote a decrease in phospholipid biosynthesis rates and therefore will decrease or inhibit membrane biogenesis-dependent cell proliferation. To test this hypothesis, we transiently transfected MDA-MB231 cells to overexpress either Fra-1-NA, c-Fos-NA, or both, tagged with a TQ2 fluorescent protein. At 48 h after transfection, quiescent cells were induced to re-enter growth and proliferation assessed as described under materials and methods. Overexpression of either of the NA deletion mutants alone or, both mutants together, significantly decrease the number of cells at 30 h after the mitotic stimulus as compared to controls (Figure 4A). Similar experiments were performed using the 4T1 mouse mammary carcinoma cell line showing similar results (Figures 4B,C). The presence of the pTQ2 vector did not significantly alter 4T1 (Figures 4B,C) or MDA-MB231 cell proliferation as compared to non-transfected cells.

### The N-terminus Domains of Fra-1 and c-Fos Inhibit Breast Tumor Growth *in vivo* in a Balb/c Mice Tumor Model

**Figure 5:**
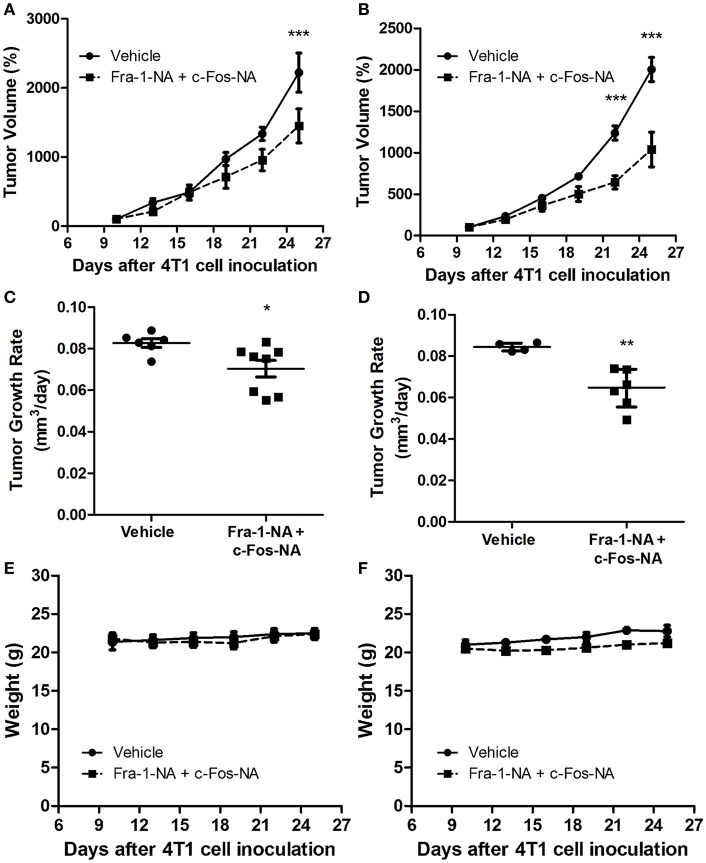
Fra-1-NA plus c-Fos-NA inhibit breast tumor growth. Mice bearing one tumor received an intratumoral injection of liposomes containing Fra1-NA plus c-Fos-NA. Peptide administered: 5 injections containing 4.5 nmol per injection (dashed line) or vehicle (continuous line) **(A,C,E)** or 9 nmol per injection **(B,D,F)** delivered three times a week for 15 days. In **(A,C,E)**, control group: *n* = 6; treated group: *n* = 8; in **(B,D,F)**, control group: *n* = 4; treated group: *n* = 6. **(A,B)** Tumor volumes were measured at 3-day intervals during the treatment period. Results are expressed as the percent volume taking the volume of the tumor at 10 days of inoculation as 100%. ^***^p < 0.001 (Two-way ANOVA, Bonferroni post-test). **(C,D)** Tumor growth rates were calculated for each group at the end of the treatment as the slope of the curve obtained by plotting the log_10_ of the tumor volume in function of the days of treatment. Horizontal bars represent mean **(C)** or median **(D)** values according to the distribution of the data (parametric and non-parametric ANOVA, respectively) ^*^p < 0.05; ^**^p < 0.01. **(E,F)** Body weight of animals during the treatment period.

As both Fra-1-NA and c-Fos-NA interfere with the proliferation of breast tumor cells in culture, we verified if these deletion mutants were capable of interfering with breast tumor growth *in vivo*. For this, the 4T1 breast tumor model was used (32). As indicated under Materials and Methods, two protocols were used for treatment, one with a 60 μg dose of 30 μg each of Fra-1-NA plus c-Fos-NA per injection (4.5 nmol per injection) and the other with a 120 μg dose of 60 μg of each peptide per injection (9 nmol per injection). After 15 days of treatment with both doses of Fra-1-NA- plus c-Fos-NA-containing liposomes, a significant reduction in tumor volume and tumor growth rate was found as compared to control tumors (Figures 5A,C: low dose; Figures 5B,D: high dose). However, by day 15 of treatment (day 25 post inoculation), control tumors reached a tumor volume ~50% larger than those treated with the lowest dose of both N-terminus deletion mutants whereas they almost doubled the size of those treated with the higher dose. No significant differences were observed in the body weight between groups (Figures 5E,F).

**Figure 6:**
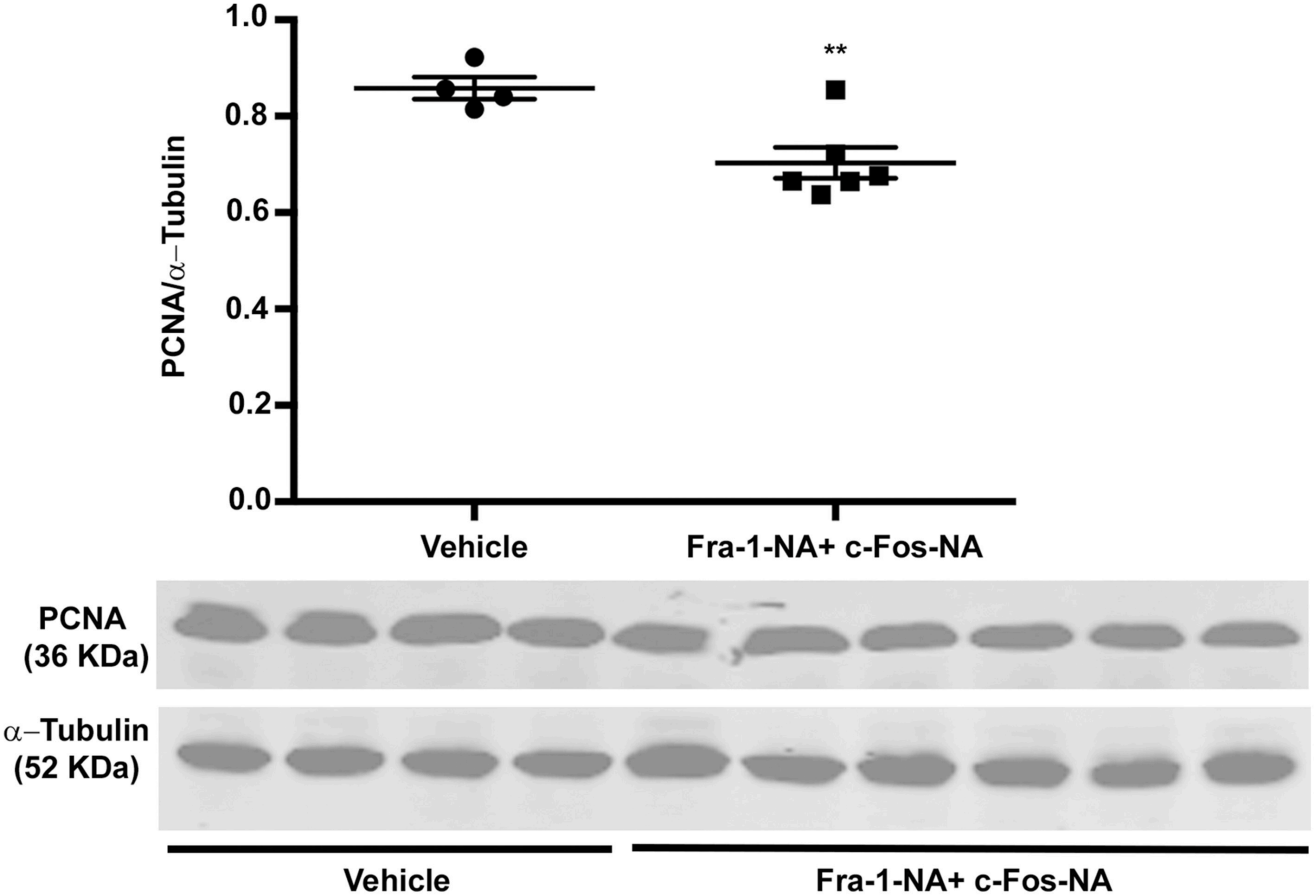
Fra-1-NA plus c-Fos-NA treatment decreases PCNA protein levels. Protein extracts prepared from the tumors excised from tumor-bearing Balb/c mice treated or not with Fra-1-NA plus c-Fos-NA were subjected to western blot analysis to examine PCNA and α-tubulin (as loading control) levels as described under Materials and Methods. PCNA and α-tubulin levels of all the tumors of the high dose treatment experiment with their controls are shown. Scatter dot plot represents the densitometric quantification and horizontal bars the mean ± SEM (Student *t*-test). ^**^p < 0.01. Control group: *n* = 4; treated group: *n* = 6.

To evaluate the proliferating status of the treated and control tumors, once tumors were excised from the animals of the high dose experiment, protein extracts were prepared from the tumors and proliferating cell nuclear antigen (PCNA) protein levels determined by western blot. Fra-1-NA plus c-Fos-NA-treated tumors showed a significant decrease in PCNA protein levels in comparison to the controls (Figure 6). The lowest PCNA levels in individual samples were found in the tumors with the lowest growth rate (data not shown), indicating a good correlation between the amount of PCNA found and the tumor growth rate calculated for each tumor.

**Figure 7:**
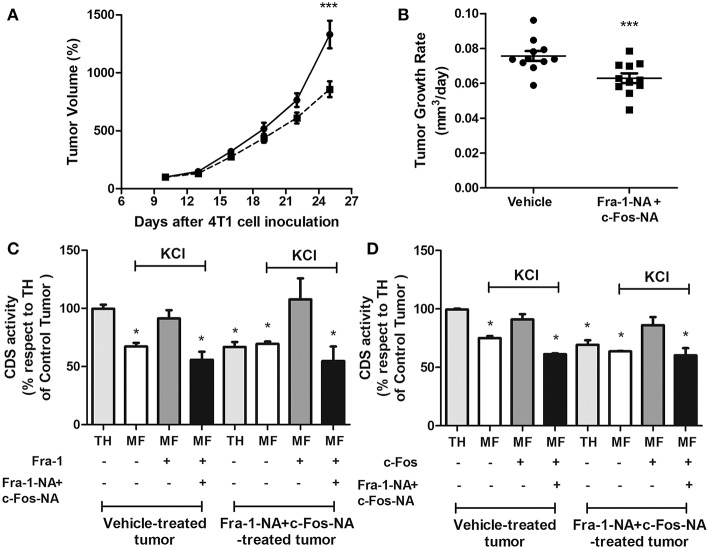
When grown in the same animal, Fra-1-NA+c-Fos-NA treatment impairs tumor growth and CDS activity. Mice bearing two tumors received intratumoral injections of liposomes containing equal concentrations of Fra1-NA plus c-Fos-NA into one tumor and empty liposomes into the other. Peptide administered: 5 injections containing a total of 9 nmol of Fra-1-NA plus c-Fos-NA peptides per injection (dashed line) or vehicle (continuous line) delivered three times a week for 15 days. **(A)** Tumor volumes were measured at 3-day intervals during the treatment period. Results are expressed as the percent volume taking the volume of the tumor at 10 days of inoculation as 100%. ^***^p < 0.001 (Two-way ANOVA, Bonferroni post-test) **(B)** Tumor growth rates were calculated for each group at the end of the treatment as the slope of the curve obtained by plotting the log_10_ of the tumor volume in function of the days of treatment. Horizontal bars represent mean values. ^***^p < 0.001 (Paired *t*-test). Mice examined: *n* = 11. **(C,D)** CDS activity was measured in TH (light gray bars) and the microsomal fraction (MF) stripped with KCl 1M (white, dark gray and black bars) from vehicle-treated tumors and Fra-1-NA plus c-Fos-NA-treated tumors of two mice. Recombinant wild type Fra-1 **(C)**, recombinant wild type c-Fos **(D)** and/or Fra-1-NA + c-Fos-NA **(C,D)** were added to the reaction as indicated in the figure. Experiments were performed in triplicate; bar graphs represent the mean ± SEM. All experimental situations were compared to TH from the non-treated samples. ^*^p < 0.05 (One-way ANOVA, Dunnett post-test).

To evaluate if the Fra-1-NA plus c-Fos-NA-treatment was triggering a systemic effect on tumor growth, assays were performed inoculating 4T1 cells into both flanks of each mouse and 10 days later, one of the two tumors generated was treated with the high dose protocol whereas the other was treated with vehicle. Consistently, tumors treated with the deletion mutants grew to a significantly smaller tumor volume and evidenced a decreased tumor growth rate when compared with the contralateral control tumor (Figures 7A,B). Once excised from the animals, paired control and treated tumors from two mice were used to evaluate total CDS activity. In both cases, CDS activity measured in TH of the vehicle-treated tumor was significantly higher than that obtained in TH of the paired Fra-1-NA plus c-Fos-NA-treated tumor from the same mouse (light gray bars in Figures 7C,D). Furthermore, when these tumor samples were stripped of associated proteins with 1M KCl and subjected to subcellular fractionation by ultracentrifugation, CDS activity in the microsomal fraction (MF) was no longer different in the control as compared to the treated tumors (white bars in Figures 7C,D). As expected, the addition of recombinant Fra-1 (dark gray bars in Figure 7C) or c-Fos (dark gray bars in Figure 7D) activated CDS but if in addition to the recombinant Fra-1 or c-Fos proteins, the N-terminus deletion mutants were included in the reaction, CDS activity decreased to similar levels of those obtained in the MF treated with KCl 1M of each condition (black bars in Figures 7C,D).

## Discussion

Most studies regarding c-Fos and Fra-1 are related to their role as transcription factors. However, both proteins also activate phospholipid synthesis by a non-genomic mechanism (35). In breast tumor models, c-Fos and Fra-1 activate phospholipid synthesis and both do so through a mechanism that is independent of their genomic activity (20). We previously showed for c-Fos that its NA is implicated in the physical association with the enzymes it activates whereas its BD is essential for activation to occur (21, 26). Herein, we demonstrate that in order to activate phospholipid synthesis in an invasive triple negative breast tumor cell model, the MDA-MB231 cell line, Fra-1, and c-Fos physically associate with CDS1 and enhance total CDS activity. By contrast, neither protein associates with or activates PIS. Both proteins associate with the enzyme they activate through their NA domain and activate it through their BD domain. Furthermore, in the presence of both NA deletion mutants, colocalization of full-length Fra-1 and c-Fos with the ER marker calreticulin decreases.

Of all the Fos family members, Fra-1 and c-Fos share the highest homology, especially on their basic domains: key for lipid synthesis activation (20). Although homology between their N-terminus domains reaches only a 34%, the *in silico* predicted disorder shows high order correlation in the central region of these peptides (Supplementary Figure 3), suggesting that these ordered regions are relevant for the oncoprotein-enzyme interaction.

We hypothesized that the NA domains of Fra-1 and c-Fos act as negative dominants that physically associate with enzymes involved in the phospholipid synthesis pathway but without activating them. Consequently, no proliferation can be expected if activation of membrane biogenesis is impaired. Indeed, the overexpression of Fra-1-NA, c-Fos-NA or both together, significantly impairs MDA-MB231 and 4T1 cell proliferation in comparison with control cells or cells transfected with the empty vector pTQ2. Other authors and our previous reports have shown that the silencing of only Fra-1 or c-Fos does not significantly affect MDA-MB231 cell proliferation (18, 20). However, silencing both proteins with siRNA or blocking their cytoplasmic activities using specific antibodies, inhibits MDA-MB231 cell proliferation (20). We inhibit tumor cell proliferation by decreasing or blocking the cytoplasmic function of Fra-1 and c-Fos without affecting their nuclear function as AP-1 transcription factors because both deletion mutants examined, lack the AP-1 dimer formation domain (LZ) of the C-terminus of these proteins and the recognition domain for their nuclear translocator system (BD) (36, 37).

Fra-1 and c-Fos are overexpressed in breast tumor tissues as compared with their undetectable levels found in normal cells (20) with a direct relationship reported for the cytoplasmic expression of Fra-1 and malignancy in breast tumor (38). Furthermore, Fra-1 mRNA is overexpressed in triple-negative breast tumors, compared to breast tumors positive for estrogen or progesterone receptors (39). Fra-1 and c-Fos overexpression together with PCNA immunoreaction are clearly observed in tumor samples, whereas PCNA immunoreactivity and c-Fos and Fra-1 expression levels are almost undetectable in the non-pathological counterparts (20). Herein, the inhibitory effect on proliferation of both NA deletion mutants were evident both *in vitro* and *in vivo* in the 4T1 breast tumor model. After the intratumoral delivery of liposomes containing Fra-1-NA plus c-Fos-NA, a significant decrease in tumor size and tumor growth rate was observed. This response seems to be due to a direct effect of the peptides rather than to a stress or systemic effect on the animal since when two tumors were induced in a single mouse, the one treated with Fra-1-NA plus c-Fos-NA showed a decrease in tumor volume, tumor growth rate and CDS activity as compared to its own control treated with empty liposomes in the same mouse. In addition, PCNA protein levels in the excised tumors from the treated group showed lower expression levels than the control, evidencing that treated tumors are less proliferative. Two different doses were tested in these experiments, and, although in both cases a decrease in tumor growth rate was observed, the highest concentration was more effective. It should be mentioned that the doses assayed were empirically determined since, although previous data on cancer treatment with proteins is available, mostly antibodies [reviewed in Scott et al. (40)], no previous reports were found using the peptides evaluated herein.

These results highlight the potential use of Fra-1-NA and c-Fos-NA as a new therapeutic strategy. Other authors demonstrated that Fra-1 plays a key role in mediating the transition to and in maintaining the population of breast cancer stem cells and also observed from a compendium of clinical data sets, that high Fra-1 mRNA expression correlates with a poor, metastasis-free survival (18). In this context, further studies are necessary to evaluate if the N-terminal deletion mutants are also capable of inhibiting the proliferation of breast cancer stem cells.

The therapy proposed in this manuscript using c-Fos plus Fra-1 deletion mutants has some advantages. First, Fra-1 and c-Fos overexpression in breast tumor tissues in comparison with undetectable levels in normal tissue confers *per se* high tumorigenic tissue specificity because the deletion mutants will interfere only with the activity of these proteins in cells overexpressing their full-length versions. Moreover, these peptides should not interfere with the canonical function of the AP-1 transcription factors in normal cells since they do not contain the domain required for AP-1 dimerization of full-length c-Fos and Fra-1. The therapeutic use of peptides and proteins is recognized as being highly selective for their *in vivo* targets resulting in exceptionally high potencies of action and, at the same time, relatively safe and well-tolerated with few off-target effects (41, 42).

The application of these mutants as an adjuvant therapeutic strategy to treat malignant breast tumors is promising and, since other tumors such as brain, lung, liver, cervical, bladder also show over expression of one or both of these proteins, it is expected that this future therapeutic strategy will be readapted or slightly modified to inhibit the growth and progression of all tumors overexpressing Fra-1 and/or c-Fos.

## Data Availability

The datasets generated for this study are available on request to the corresponding author.

## Ethics Statement

This study was carried out in accordance with the recommendations of the ethics committee of experimental protocols for the use of animals (Facultad de Ciencias Químicas, Universidad Nacional de Córdoba). The protocol was approved by the ethics committee of experimental protocols for the use of animals (Facultad de Ciencias Químicas, Universidad Nacional de Córdoba. Resolution N° 2102).

## Author Contributions

AR: conception and design of the study, experimental work and acquisition of data, analysis and interpretation of data, manuscript writing, and final approval of the manuscript. CP: conception and design of the study, experimental work and acquisition of data, analysis and interpretation of data, revising draft critically, and final approval of the manuscript. BC: conception and design of the study, analysis and interpretation of data, manuscript writing, and final approval of the manuscript.

### Conflict of Interest Statement

The authors declare that the research was conducted in the absence of any commercial or financial relationships that could be construed as a potential conflict of interest.
