# Supplementary material for: Fra-1 and c-Fos N-Terminal Deletion Mutants Impair Breast Tumor Cell Proliferation by Blocking Lipid Synthesis Activation
Source: Front Oncol. 2019 Jun 19;9:544. doi: 10.3389/fonc.2019.00544 (PMC6593343; doi:10.3389/fonc.2019.00544)
Supplement: Supplementary file 1 [file Data_Sheet_1.pdf]

Supplementary Material

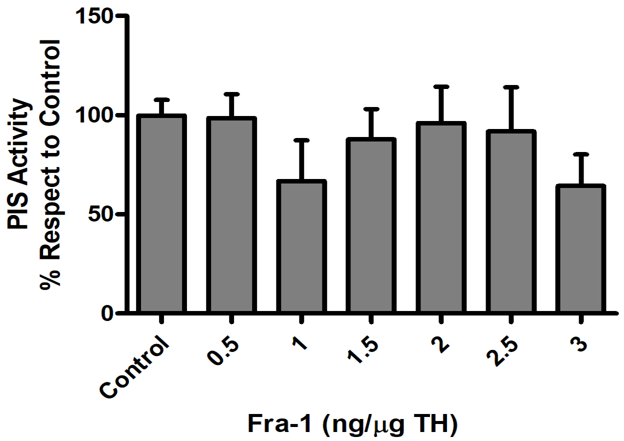

Supplementary Figure 1. Fra-1 does not activate PIS

Total homogenate (TH) from quiescent MDA-MB231 cells was used as enzyme source to evaluate PIS activity in the presence of increasing concentrations of recombinant Fra-1 at 15 min of incubation; concentrations assayed ranged between 0 and 3 ng of Fra-1/ µg of TH. None of the Fra-1 concentrations evaluated significantly modified PIS activity. Results are the mean enzyme activity ± SEM of three independent experiments performed in triplicate (One-way ANOVA).

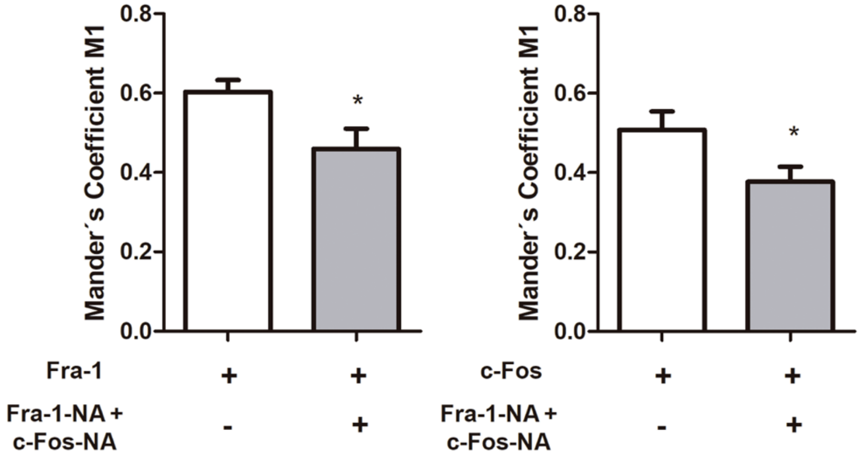

Supplementary Figure 2. Fra-1 and c-Fos colocalization with calreticulin decreases in the presence of the N-terminus Fra-1 and c-Fos deletion mutants

MDA-MB231 cells were co-transfected with plasmids that codify for pEGFP-Fra-1 or pEGFP-c-Fos and Fra-1-NA-HA + c-Fos-NA-HA or the corresponding empty vector. The endoplasmic reticulum (ER) marker calreticulin was detected with a primary antibody. Mander´s colocalization

coefficient M1 was used to analyze the proportion of Fra-1 or c-Fos that correlates with ER signal in the absence or presence of Fra-1-NA and c-Fos-NA deletion mutants. Results are expressed as the mean ± SEM of two independent experiments analyzing at least 20 cells per condition. *$p<0.05$ (Student t-test).

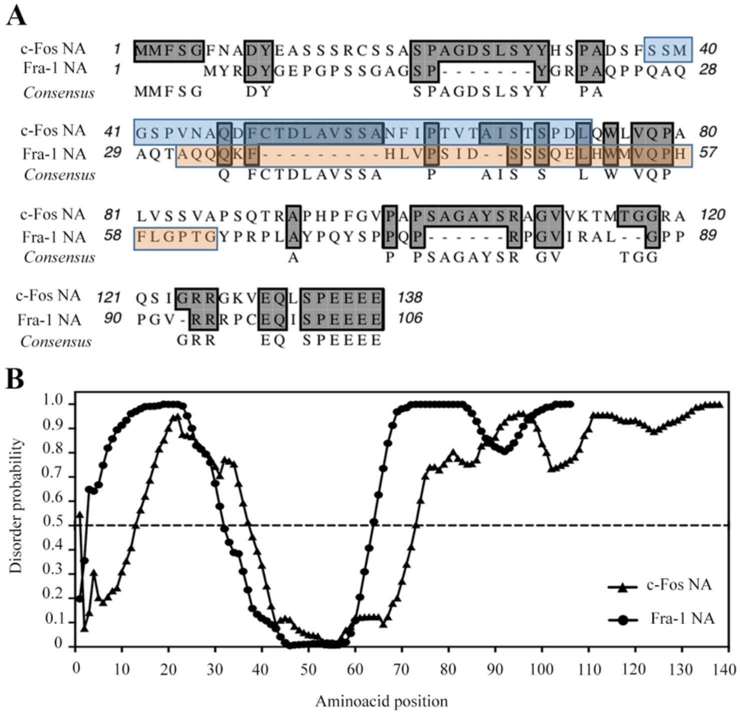

Supplementary Figure 3. In silico analysis comparison of Fra-1-NA and c-Fos-NA

(**A**) Amino acid sequence alignment of c-Fos-NA and Fra-1-NA using the MUSCLE algorithm. Conserved amino acids are indicated in bold letters (alignment performed using MacVector software). (**B**) Disorder probability comparison between c-Fos-NA (▲) and Fra-1-NA (●) using PONDR. The y axis shows the disorder probability; values below 0.5 correspond to putative ordered zones delimited by the black dashed line. The x axis indicates the amino acid position, both deletion mutants share an internal ordered region: c-Fos-NA ordered amino acids: 38-73; Fra-1-NA ordered amino acids: 32-63. In **A**, amino acids belonging to the putative ordered domain are highlighted in blue for c-Fos-NA and in pink for Fra-1-NA.
